# Effects of Bilingualism on Executive Function of Children with Neurodevelopmental Disorders: A Scoping Review

**DOI:** 10.3390/children12091247

**Published:** 2025-09-17

**Authors:** Hoi Kwan Yuen, Haoyan Ge, Caicai Zhang, Yuen Ting Wong, Eva Y. W. Chan, William W. N. Tsang, Catherine M. Capio

**Affiliations:** 1 Department of Physiotherapy, Hong Kong Metropolitan University, Hong Kong SAR, China; hkyuen@hkmu.edu.hk (H.K.Y.); yutwong@hkmu.edu.hk (Y.T.W.); eywchan@hkmu.edu.hk (E.Y.W.C.); wntsang@hkmu.edu.hk (W.W.N.T.); 2 School of Education and Languages, Hong Kong Metropolitan University, Hong Kong SAR, China; hge@hkmu.edu.hk; 3 Department of Language Science and Technology, Hong Kong Polytechnic University, Hong Kong SAR, China; caicai.zhang@polyu.edu.hk

**Keywords:** bilingualism, executive function, neurodevelopmental disorders, autism spectrum disorder, cognitive development, working memory, cognitive flexibility, inhibitory control, language development, developmental disabilities

## Abstract

**Highlights:**

**What are the main findings?**

- Compared with their monolingual peers, bilingual children with ASD show advantages in working memory, cognitive flexibility, and inhibitory control on performance-based tasks.
- The evidence is limited to ASD, with no studies found on other neurodevelopmental disorders, such as ADHD or dyslexia, highlighting a research gap.
- Inconsistencies exist between performance-based and parent-reported executive function measures, with bilingual participants performing better in laboratory tasks.

**What is the implication of the main finding?**

- Bilingualism may be beneficial for specific executive function skills in children with ASD, suggesting potential complementary value to traditional interventions.
- Further research is needed to explore the relationship of bilingualism with executive function across diverse neurodevelopmental disorders to inform clinical and educational practices.

**Abstract:**

**Background:** Children with neurodevelopmental disorders (NDDs) commonly experience executive function (EF) impairments that impact daily life and academics. While bilingualism has generally been associated with cognitive advantages in typically developing (TD) children, its relationship with EF in children with NDDs remains unclear and represents a critical knowledge gap for families and clinicians considering bilingual exposure in these populations. **Methods:** For this scoping review, we searched PubMed, ProQuest, CogNet, PsycINFO, Scopus, ERIC, Embase, CINAHL, Linguistics Abstracts Online, and Google Scholar for studies published between database inception and December 2024, without language restrictions. We included quantitative, qualitative, and mixed-methods studies that (i) involved participants aged 4–12 years with diagnosed NDDs; (ii) examined children with bilingual language exposure; (iii) employed validated instruments for measuring cognitive or executive function; (iv) presented original empirical findings; and (v) were published in English. We excluded studies lacking comparisons between groups and longitudinal studies. Data on study characteristics, participants, EF assessments, and main findings were extracted. This study is registered with OSF Registries. **Findings:** Fifteen cross-sectional studies met the inclusion criteria, all of which focused exclusively on children with autism spectrum disorder (ASD), with no studies examining other NDDs. The studies involved 982 children with ASD (463 monolingual; 404 bilingual) and 644 TD children. Most studies (n = 11) revealed that, compared with monolingual children with ASD, bilingual children with ASD demonstrated advantages in working memory, cognitive flexibility, and inhibitory control on performance-based tasks. However, findings were inconsistent for spatial inhibition tasks, and parent-reported measures sometimes failed to detect bilingual-related differences. **Interpretation:** Bilingualism is associated with specific EF benefits for children with ASD, adding to evidence that questions longstanding concerns about the negative impacts of bilingual exposure in NDD populations. The evidence suggests that bilingual exposure could potentially serve as a complementary approach to traditional interventions for addressing EF impairments in children with ASD, although this evidence is limited to cross-sectional designs and requires further studies. However, the exclusive focus on ASD limits generalisability across the broader spectrum of NDDs. Further research is needed across diverse NDD populations employing comprehensive, multi-method EF assessments that combine performance-based tasks with parent-reported measures to better inform parenting, clinical, and educational practices.

## 1. Introduction

Children with neurodevelopmental disorders (NDDs) experience challenges and variability in their daily lives, but these challenges can be addressed through intervention programmes and environmental accommodations. NDDs refer to a group of conditions with onset during the developmental period [1]. According to the Diagnostic and Statistical Manual of Psychiatric Disorders (DSM-5), NDDs include a range of conditions, such as cerebral palsy (CP), developmental coordination disorder (DCD), autism spectrum disorder (ASD), intellectual disability (ID), dyslexia, and attention deficit-hyperactivity disorder (ADHD) [2]. Although each NDD is unique in its clinical presentation, they share a common thread of diversity in cognition, communication, behaviour, and motor skills that affect daily functioning [3].

In the cognitive domain, challenges associated with executive function (EF) are pervasive issues among children with NDDs. Research consistently shows that children with conditions such as ADHD, ASD, and DCD exhibit moderate to large variabilities in EF domains, including working memory, cognitive flexibility, inhibition, and planning, compared with typically developing (TD) peers [4,5]. These EF variabilities are transdiagnostic, affecting daily functioning and academic achievement, highlighting the need for targeted interventions to support EF development in this population [6,7].

### 1.1. Executive Function

EF refers to higher-order cognitive processes that include inhibitory control, working memory, and cognitive flexibility, which are necessary for goal-oriented problem solving in everyday functioning [8,9,10]. EF plays a significant role in determining a variety of educational and social outcomes and has a broad impact on quality of life. The ability to engage in the cognitive processes of EF is critical in solving the immediate, medium-term, and long-term problems that occur in daily life [11]. For instance, EF skills allow individuals to assess whether their actions are efficient or whether they should abandon actions that appear to be inefficient. A systematic review revealed that children with DCD experience challenges in inhibitory control, working memory, planning, and general EF [12]. Children with ASD also experience difficulties with cognitive flexibility [13], inhibition [14], and working memory [15]. Similarly, children with CP may exhibit variable EF, particularly in inhibition and working memory [16,17]. Children with ID experience difficulties across multiple EF domains, including working memory, cognitive flexibility, and inhibitory control [18]. EF difficulties are also well documented in children with ADHD, with deficits in inhibitory control, working memory, and cognitive flexibility [19,20]. Additionally, children with dyslexia also show EF deficits, particularly in tasks requiring the inhibition of distractors and the sequencing of events [21,22]. EF difficulties across the three domains among the NDDs are summarised and mapped in Table 1.

The transdiagnostic nature of EF impairments across NDDs is further complicated by the high rates of comorbidity between these conditions. Research has consistently demonstrated substantial overlap between different NDDs, with children frequently presenting with multiple concurrent diagnoses [23,24]. For example, studies report comorbidity rates of 50–70% between ADHD and DCD [25,26], whereas approximately 28–44% of children with ASD also meet the criteria for ADHD [4,27]. Similarly, high comorbidity rates exist between dyslexia and ADHD [28] and between intellectual disability and other NDDs [29,30]. These overlapping presentations suggest shared underlying mechanisms and pathways, making it crucial to examine interventions that could address common EF deficits across the spectrum of NDDs rather than focusing on isolated conditions [31]. A review examining bilingualism effects across different NDDs may reveal key knowledge that would be missed in condition-specific reviews and help us better understand the inter-relationships between these conditions, particularly given their shared EF vulnerabilities and frequent co-occurrence.

### 1.2. Bilingualism

Bilingualism is defined as the ability to use two or more languages for communication, regardless of proficiency level or age of acquisition [32,33]. This definition encompasses a wide spectrum of bilingual experiences, from balanced bilinguals who demonstrate equal proficiency in both languages to those with varying degrees of competence across their languages [33,34].

Investigating bilingualism in the NDD population is particularly important for several reasons. First, bilingualism represents the norm globally, with more than half of the world’s population being multilingual [35]. This exposure is often unavoidable, making it essential to understand how bilingualism impacts cognitive development in this population. Second, bilingualism offers numerous benefits beyond cognitive advantages, including enhanced cultural identity, improved social connections, expanded educational and career opportunities, and stronger family bonds when multiple languages are spoken within the household [36].

The relationship between bilingualism and enhanced cognitive functioning has been the subject of considerable research and debate, with substantial literature reporting both positive and null effects in TD populations. Some studies have shown that bilingual individuals outperform monolingual individuals on various EF tasks [34,37,38], whereas others have failed to find consistent advantages or have reported no significant differences [39,40,41]. These discrepancies may arise from methodological concerns, such as publication bias, task impurity, and different measurement tools used across studies. The underlying mechanism for a bilingual-related difference in cognitive function is hypothesised to be based on neuroplasticity, that is, the brain’s adaptivity and formative ability gained from experiences managing and switching two languages in different settings [35]. It is believed that the cognitive advantage arises from the additional demands placed on domain-general cognitive processes when managing two languages. Thus, bilingualism may aid in EF development. However, the evidence regarding bilingual cognitive advantages remains controversial and mixed. While some studies report significant benefits, others have failed to find consistent advantages, with some even reporting no significant differences in EF among bilingual children [39,40,41]. For children with NDDs who have EF impairments, however, the evidence supporting the potential role of bilingualism has yet to be synthesised. Moreover, existing language and cognitive impairments lead parents and professionals to wonder whether exposure to more than one language increases the burden on children with NDDs [42,43].

### 1.3. Current Review

Previous reviews have examined the relationship between bilingualism and EF, but they have focused primarily on TD children, often reporting cognitive benefits [44,45]. Systematic reviews have shown that bilingual children tend to exhibit advantages in terms of cognitive flexibility, inhibitory control, and set-shifting abilities [44,46]. Conversely, other studies have failed to find consistent benefits from bilingualism, with some reporting no significant differences or even potential disadvantages in EF among bilingual children [39,40,41]. These discrepancies may stem from considerable heterogeneity in study designs, sample characteristics, language proficiency levels, and EF assessment tools.

When it comes to children with NDDs, the evidence base is unclear, given the small number of studies focusing on this population group. Such a lack of clarity poses challenges for clinicians, educators, and families seeking guidance on bilingual exposure for children with NDDs. Without a clear understanding of how bilingualism interacts with EF development in this population, it is difficult to formulate evidence-based recommendations or intervention strategies. Therefore, a scoping review that maps the existing evidence on bilingualism and EF across a range of NDDs is warranted. Such a review will not only identify the scope and nature of the available evidence but also highlight methodological gaps and inform future research priorities. Furthermore, by consolidating current knowledge, this work aims to support clinicians and educators in making informed decisions and contributing to the development of tailored interventions that consider bilingualism as a potential factor influencing cognitive outcomes in children with NDDs. A scoping review is deemed an appropriate evidence synthesis, as it provides a broad and flexible framework to explore diverse study designs, populations, and outcome measures and to synthesise evidence in a way that can guide both research and practice [47,48]. Our objective is to evaluate the scope of research on the relationship between bilingualism and EF in children with NDDs, specifically by identifying the NDD groups that have been studied; describing the study designs and methodologies used to examine bilingualism and EF domains; and synthesising existing evidence to inform future research and clinical practice.

## 2. Methods

This review was registered with OSF Registries (DOI: 10.17605/OSF.IO/Z8YNT). The review methodology followed the established scoping review framework developed by Arksey and O’Malley [49] and the refined guidelines from the Joanna Briggs Institute (JBI) [50]. Five core phases were implemented: (1) formulating the research question, (2) identifying relevant literature, (3) study selection, (4) data extraction, and (5) summarising and reporting the findings. The optional sixth phase involving stakeholder consultation [49] was not implemented for this review because of the exploratory nature of this initial evidence mapping, and it was not necessary to address the research questions. Nevertheless, we acknowledge that the omission of stakeholder consultation may limit the applied relevance of findings. In accordance with the JBI recommendations for scoping reviews [50], quality assessment or risk of bias evaluation was not performed.

### 2.1. Formulating the Research Question

The Population, Concept, and Context (PCC) framework recommended for scoping reviews [51] was applied to develop the following research question: What is the state of evidence relating bilingualism with EF outcomes (concepts) in children diagnosed with NDDs (population) within developmental and educational contexts (context)? The scoping review addresses the following research objectives:

1. To identify the specific NDD groups that have been studied in relation to bilingualism and EF.
2. To determine the study design and methodology of studies that explored the relationship between bilingualism and EF domains in children with NDDs.
3. To synthesise existing evidence of the relationship between bilingualism and EF domains to guide future investigations and clinical applications.

### 2.2. Identifying the Relevant Literature

We conducted a systematic search of peer-reviewed literature encompassing quantitative, qualitative, and mixed-methods research designs. The initial search was performed in December 2024, with no time restrictions applied. Our search encompassed multiple electronic databases: PubMed, ProQuest, CogNet, PsycINFO, Scopus, ERIC, Embase, CINAHL, and Linguistics Abstracts Online. Grey literature was identified through Google Scholar and targeted unpublished research, including dissertations, theses, and conference materials. We systematically reviewed the first 200 Google Scholar results for each search combination.

Our search was supplemented by backwards and forward citation searches of key included studies to ensure broad coverage of the relevant literature. The search terms were combined via Boolean logic (“AND”, “OR”, “*”) to create comprehensive search strings. Drawing from our PCC framework, we developed the following search strategy: (“bilingualism” OR “L1 L2” OR “first language” OR “second language”) AND (“executive function” OR “cognitive function”) AND (“children” OR “kids”) AND (“neurodevelopmental disorder” OR “developmental disability” OR “special education needs”). The search results were processed through Covidence [52], a systematic review platform.

### 2.3. Study Selection Process

**Figure 1:**
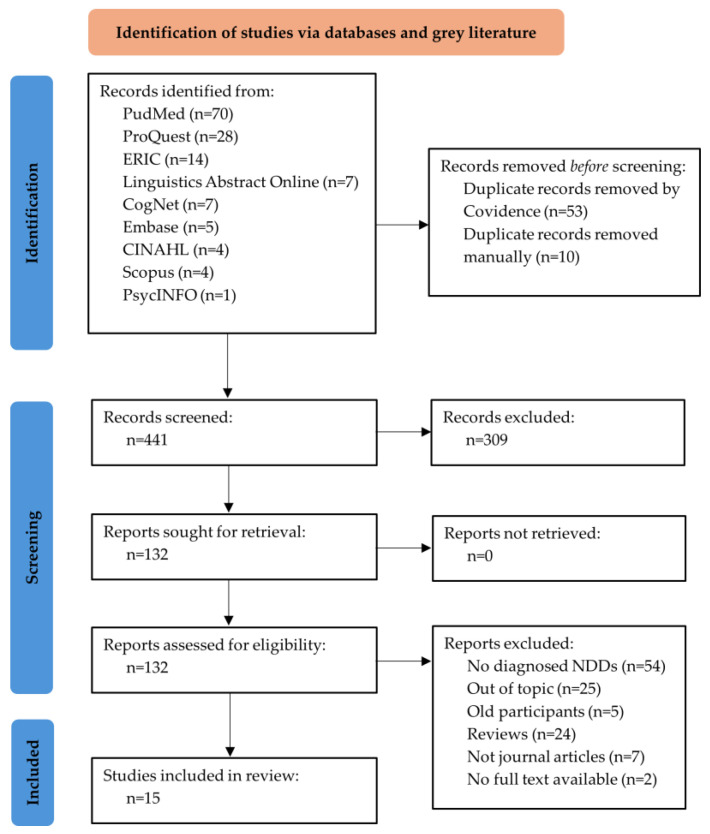
PRISMA 2020 flow diagram [53].

Our study selection process (Figure 1) followed the Preferred Reporting Items for Systematic Reviews and Meta-Analyses (PRISMA) guidelines [53] and the PRISMA Extension for Scoping Reviews (PRISMA-ScR; [48]). Two independent reviewers (first author, fourth author) conducted the selection process using Covidence systematic review tool [52], which facilitated duplicate removal and systematic screening through automated features and collaborative review functions.

Following duplicate removal, initial title screening eliminated clearly irrelevant studies. Two reviewers independently screened the titles and abstracts of all retrieved studies for potential eligibility, and irrelevant studies not meeting the inclusion criteria were excluded. Studies were deemed eligible if they (i) involved participants aged 4–12 years with diagnosed NDDs, (ii) examined children with bilingual language exposure (i.e., defined as consistent exposure to or use of multiple languages in daily life), (iii) employed validated instruments for measuring cognitive or executive functioning, (iv) presented original empirical findings rather than reviews, and (v) were published in English. The restriction to English-language publications represents a limitation of this review and may have excluded relevant studies in other languages. The exclusion criteria were as follows: (i) studies lacking comparisons between groups; (ii) longitudinal studies; and (iii) preliminary reports such as protocols, conference abstracts, or incomplete dissertations.

Full-text articles of potentially relevant studies were then retrieved and independently assessed by the two reviewers. Disagreements were resolved through discussion, with a third reviewer (last author) consultation when consensus could not be reached.

### 2.4. Data Extraction Procedures

A data extraction framework was developed following the JBI scoping review protocols [50]. The following data were extracted: author(s), title, year of publication, setting, study design, participants, EF and bilingualism measures, and main findings. Data extraction was performed by one reviewer (first author) with secondary verification by the second reviewer (fourth author) to ensure accuracy and completeness. Inter-rater reliability for data extraction was assessed through independent extraction of a subset of studies, with disagreements resolved through discussion and achieving 97% agreement (calculated using percentage agreement).

### 2.5. Summarising and Reporting Findings

The extracted data were recorded in the data extraction framework and tabulated (see the Supplementary File). The findings are reported in terms of the NDD groups studied, EF assessments, and EF outcomes, as presented in the following section.

## 3. Results

### 3.1. Overview of the Studies

Our initial search yielded 504 results. After removing duplicates, we screened 441 records, from which we reviewed 132 full-text documents, and 15 papers were ultimately included (Figure 1). All of the studies employed cross-sectional designs (n = 15). Key information from each study is presented in the Supplementary Table S1. EF assessments and settings for the studies are summarised in Table 2, while the outcomes per EF domain are mapped in Table 3.

### 3.2. NDD Groups Studied

The search strategy, despite the use of broad terms to capture various NDDs, yielded 15 studies that focused exclusively on children with ASD. This represents a significant gap in the evidence base, as other conditions, such as ADHD, DCD, intellectual disabilities, and dyslexia, remain unexplored in the context of bilingualism and EF. This concentration of efforts within a single NDD condition represents both an opportunity and a significant limitation.

The study participants ranged in age from 5 to 12 years and included 982 children with ASD (n = 463 monolingual; n = 404 bilingual) and 644 TD children (n = 346 monolingual; n = 298 bilingual) for comparison. Only two studies reported their participants’ socioeconomic status (SES) [54,67], whereas two others provided information on intelligence [61,65]. No further detailed information about the participants’ characteristics was reported in other studies. Participant characteristics (such as SES and intelligence levels) were inconsistently reported across studies, limiting the ability to assess potential confounding variables. The studies examined cognitive function outcomes through comparisons of the ASD groups with the TD groups. None of the included studies reported data on linguistic distance between language pairs or examined its role, representing a notable gap in the evidence base.

### 3.3. EF Assessments

The most frequently reported EF assessment in the reviewed studies was the two-back task (Kirchner, 1958) [69] (n = 6), which assesses working memory. Global-local tasks [69] were used in four studies to examine attention and cognitive flexibility (n = 4). Four studies employed the Behaviour Rating Inventory of Executive Function (BRIEF) [70] as a parent-reported measure of EF (n = 4). Three studies used the Simon task [71] for inhibitory control assessment (n = 3). The Comprehensive Executive Function Inventory (CEFI) [72] was used in two studies (n = 2), along with the Dimensional Change Card Sort (DCCS) [73] for cognitive flexibility (n = 2). One study each used the Stroop task [74] for inhibitory control, the Go/No-Go task [75] for response inhibition, the WCST for cognitive flexibility, the Flanker task [76] for attention and inhibition, digit span [77] and listening span tasks [78] for working memory, number repetition (subtest from Clinical Evaluation of Language Fundamentals, Fourth Edition (CELF-4)) [79] for phonological processing, and the SOPT [80] for working memory and executive control. A summary of the EF assessments and the settings of the studies is summarised in Table 2.

EF measures vary considerably across studies, including a mix of performance-based tasks (e.g., two-back, Simon, Flanker, global–local, DCCS) and parent-reported questionnaires (e.g., BRIEF, CEFI). This variability in task types, such as those emphasising verbal vs. spatial demands, updating vs. storage in working memory, or response-based vs. attentional inhibition, likely contributed to inconsistent findings, as different tasks may capture distinct aspects of EF and be differentially sensitive to bilingual-related differences.

### 3.4. Settings

The research was conducted across multiple countries, with the majority of studies occurring in Greece (n = 6) and Canada (n = 6), followed by the United States (n = 3), the United Arab Emirates (n = 2), and Japan (n = 1).

### 3.5. Executive Function Outcomes

#### 3.5.1. Working Memory

Performance-based tasks showed some advantages for bilingual children with ASD in working memory domains. Compared with their monolingual peers, bilingual children with ASD demonstrated better performance in two-back tasks, with higher accuracy and faster response times [54,55,56,57]. Parent-reported data from BRIEF indicated that bilingual children with ASD had fewer reported problems with working memory than monolingual children with ASD did [63]. Additional parent reports suggested lower impairment in self-monitoring for bilingual youth with ASD than for their monolingual peers [62,63].

However, several tasks, including span tasks (digit span, listening span, and number repetition), which primarily assess storage capacity, did not significantly differ between monolingual and bilingual children with ASD. Additionally, one study using parent-reported measures reported no significant difference in working memory between groups [65]. This pattern of mixed results suggests that bilingual-related differences may be specific to updating rather than capacity components of working memory and may not be consistently detected across all assessment methods.

#### 3.5.2. Inhibition and Interference Control

Bilingual children with ASD demonstrated shorter latency (faster response times) for incongruent trials on the Stroop task, suggesting better inhibition efficiency than monolingual children with ASD [59]. They also showed advantages in cognitive control when they needed to inhibit local details to focus on the global picture in global–local tasks [55,56,57], whereas monolingual children with ASD experienced significantly greater switching costs in trials, necessitating a local-to-global shift [57]. Parent-reported data from the BRIEF indicated that bilingual children reported lower inhibition difficulties than monolingual children with ASD [64,66]. Bilingual children also showed advantages in the CEFI, including interference control [64].

However, other EF assessment tasks, such as the Simon task and Flanker task, showed no significant differences between monolingual and bilingual ASD groups in terms of accuracy or reaction time for interference control [59,60,61], suggesting that the type of inhibition measured by different tasks may have inconsistent results [61]. The results varied significantly by task type, with a notable pattern of inconsistent findings across different inhibitory control paradigms. Global-local tasks (n = 4), which require attentional control and shifting between different levels of visual processing, consistently favoured bilingual children. However, spatial inhibition tasks, such as the Simon task (n = 3) and the Flanker task (n = 1), which require response-based inhibitory control, were not significantly different between the monolingual and bilingual groups. These inconsistent findings reduce the strength of conclusions and highlight that bilingual-related differences in inhibitory control are not universal across all types of inhibition tasks but are task-specific.

#### 3.5.3. Shifting and Cognitive Flexibility

Bilingual children with ASD demonstrated significant advantages in assessment tasks investigating set-shifting abilities. Compared with monolingual children with ASD, these children performed surprisingly well on the DCCS task [58,66,67]. A study by [67] even reported that bilingual children with ASD performed similarly to their TD peers.

Parent-reported measures for shifting have shown mixed results. Some studies reported lower parent-reported problems with shifting for bilingual youth with ASD [63], which was evident even after controlling for covariates such as gender, socioeconomic status, and intelligence [63,64,66]. However, other parent reports from the BRIEF or CEFI did not consistently find significant advantages for bilingual children with ASD in flexible switching [58,60,61,64,65,68], showing discrepancies between performance-based and parent-reported measures. This discrepancy may reflect differences in ecological validity, where laboratory tasks capture specific cognitive abilities under controlled conditions but may not capture real-world EF demands. Moreover, there is potential perceptual bias in parent reports, where caregivers’ expectations about bilingualism or cultural factors may influence their ratings of everyday executive functioning.

#### 3.5.4. Summary of the Findings

The evidence suggests that bilingualism may be associated with advantages in EF across multiple domains in children with ASD (see Table 3 for a summary). However, the strength and consistency of these effects vary across studies and specific EF components. The relationship appears to be complex and potentially influenced by factors such as the degree of bilingual exposure and the assessment methodologies employed. Controlling for confounders was inconsistent across studies, with some controlling for factors such as gender, SES, and intelligence levels [63,64,66], whereas others did not explicitly report or consistently control for these variables. This inconsistency in controlling for potential confounders may contribute to variability in findings and limit comparability across studies.

## 4. Discussion

### 4.1. NDD Populations

The most striking finding from this review is the exclusive focus on ASD among the fifteen identified studies. Despite the use of broad search terms to capture various NDDs, no studies have examined the relationship between bilingualism and EF in children with DCD, specific learning disorders, intellectual disabilities, or dyslexia [2]. This concentration of research efforts within a single NDD condition represents both an opportunity and a significant limitation, particularly given that EF impairments are transdiagnostic and affect daily functioning and academic achievement across the spectrum of NDDs [6,7]. This finding highlights an opportunity for researchers to pursue further research, as the gap in evidence warrants studies that examine the relationship of bilingualism with EF in other NDDs. Moreover, comparative studies across different NDDs are needed to determine whether the relationships of bilingualism with EF are disorder-specific or shared across conditions. Such research should also consider the impact of comorbidities that are frequently present in NDD populations (e.g., concurrent ASD and ADHD), which may complicate the interpretation of findings.

The focus on ASD may reflect several factors. First, the core characteristics of ASD that include difficulties with social communication and repetitive behaviours [81] may make the potential cognitive benefits of bilingualism particularly salient for researchers and clinicians. Second, the established relationship between EF difficulties and ASD symptoms, including documented difficulties with cognitive flexibility, inhibition, and working memory [82,83], likely motivates investigations into factors that could enhance EF abilities in this population. However, this singular focus leaves substantial gaps in our understanding of how bilingualism might affect EF across the broader spectrum of NDDs. These gaps need to be addressed, given that children with conditions such as DCD also demonstrate weaknesses in inhibitory control, working memory, planning, and general EF [12,84].

The demographic characteristics of the studied population also revealed important patterns. All the studies focused on children aged 5–12 years, with participants ranging from various linguistic and cultural backgrounds across multiple countries. However, studies examining Chinese children, which represent a significant gap in cultural and linguistic diversity, are notably absent from the research base. We note that the nature of bilingual experience can vary substantially across different linguistic combinations and cultural contexts [85]. In Hong Kong, for example, children are typically exposed to Chinese (Cantonese) as their mother tongue and primary medium of instruction in schools, whereas English is encountered through dedicated English language classes, English-medium-instruction subjects, and various social contexts [86,87]. However, no studies have examined the utilisation of this opportunity for bilingualism as a means to address EF impairments for Chinese children with ASD.

### 4.2. EF Assessment and Measures

There is a diversity of assessment tools used across studies, and inconsistencies are found when examining the pattern of results across different assessment approaches, particularly in measures related to set-shifting abilities. EF assessment tasks have consistently demonstrated bilingual-related differences in various domains, where bilingual children with ASD show advantages over their monolingual peers. However, parent-reported measures of the same cognitive domain often yield mixed or contradictory results. For example, while studies have reported better set-shifting abilities in bilingual children with ASD via experimental tasks, parent-reported measures have failed to detect these advantages [58], creating notable discrepancies between laboratory-based performance and real-world behavioural observations.

EF assessment tasks in laboratory settings may capture specific cognitive abilities under controlled conditions that optimise the expression of bilingual-related differences, whereas parent reports may reflect the complex interplay of EF skills with environmental demands, behavioural regulation, and adaptive functioning in naturalistic settings [88]. The controlled nature of assessment tasks may also reduce confounding variables that could mask bilingual effects in everyday contexts, such as varying task demands, social expectations, or situational stressors that children encounter in their daily lives. This suggests that bilingual-related differences in EF may be more apparent in certain contexts or may require specific conditions to manifest. Only one study used both experimental tasks and parent-reported measures within the same sample [58]. To address these methodological concerns, future research should adopt standardised, multi-method assessment protocols that combine performance-based tasks administered under controlled conditions, parent and teacher rating scales for ecological validity, structured observations in naturalistic settings (e.g., classroom, home), and ecological momentary assessments to capture real-time EF demands. Additionally, longitudinal studies, which are currently absent from the literature, would provide valuable insights into developmental trajectories.

### 4.3. Relationship Between Bilingualism and EFs

The current evidence reveals a complex but generally positive relationship between bilingualism and EF, providing important insights into the potential contribution of bilingual exposure to addressing EF difficulties in children with ASD.

The results in the working memory domain suggest advantages in assessment tasks, where bilingual children with ASD demonstrate better accuracy and faster response times in two-back tasks. This finding is particularly noteworthy given that working memory concerns are well documented across NDDs and significantly impact daily functioning [89] and academic achievement [90,91]. The convergence of evidence from assessment tasks and parent-reported measures strengthens the findings that bilingualism may be associated with working memory advantages. However, mixed results from some assessment tasks highlight the importance of considering task-specific factors in EF assessments. Verbal and spatial span tasks differ from two-back tasks in fundamental ways. Span tasks primarily assess working memory storage capacity and serial recall abilities, requiring participants to maintain and reproduce sequences of increasing length [92]. In contrast, two-back tasks assess working memory updating and manipulation processes, with a constant memory load but continuous updating demands [93]. Span tasks require effortful recall of entire sequences, whereas two-back tasks involve recognition-based matching of current stimuli to previously presented items [93]. These differences in cognitive demands may explain why apparent advantages of bilingualism were observed in some working memory tasks but not others, suggesting that bilingualism may specifically contribute to updating and manipulation processes rather than working memory capacity broadly. The observed effects may also be influenced by socio-cultural factors, educational background, and varying levels of language proficiency, requiring more future studies that would test mechanistic hypotheses. Additionally, the ongoing debate regarding the bilingual-related differences in TD populations warrants further exploration to determine whether these effects are specific to certain cognitive processes or shaped by broader environmental and individual differences.

There have been mixed results regarding the inhibition and interference control domains of EF. While several studies reported advantages for bilingual children with ASD in tasks requiring inhibitory control, other studies using different paradigms reported no differences with monolingual children. Bilingual children with ASD performed better in global–local tasks than in the Simon and Flanker tasks. Global–local tasks require participants to focus their attention on either the overall (global) structure of a stimulus or its individual (local) components [94]. For bilingual participants, managing two language systems requires flexible attention shifting between different levels of linguistic information [95]. Such a process likely enables bilinguals to outperform monolinguals in global–local tasks because of their enhanced attentional flexibility from managing two languages. However, the inhibition required in the Simon and Flanker tasks is more spatial and response-based [96,97], which may be considered to involve only partial overlap with the inhibition that bilinguals routinely practice. Hence, bilingual-related differences in these tasks are not observed consistently. Bilingualism likely contributes to enhanced inhibitory control, but this advantage is most pronounced in verbal or language-related contexts and does not consistently extend to spatial inhibitory control tasks such as the Simon or Flanker tasks [98].

The results concerning shifting abilities and cognitive flexibility generally revealed advantages among bilingual children, particularly in set-shifting tasks such as the DCCS. The finding that bilingual children with ASD perform similarly to TD peers in cognitive flexibility tasks, while their monolingual counterparts show greater difficulties, suggests that bilingualism may help ameliorate some of the cognitive inflexibility associated with ASD. This finding is particularly significant given that cognitive flexibility is related to adaptive functioning in children with ASD [99].

A notable methodological consideration in the current literature is that most studies examining the relationship between bilingualism and EF have relied on a binary classification of children as either monolingual or bilingual. This approach fails to capture the dynamic, fluid, and multidimensional nature of bilingual experiences, such as varying degrees of proficiency, frequency of language use, age of acquisition, context, and balance between languages [85,100]. Increasing evidence suggests that bilingualism is better conceptualised as a continuum rather than a discrete category and that recognising this complexity is critical for understanding cognitive outcomes [5,101,102]. As a result, the inconsistent findings observed across studies may partly stem from the oversimplification inherent in binary groupings. For example, Giovannoli and colleagues [44] highlighted that individual differences in language exposure, proficiency, and switching frequency can modulate executive function outcomes in bilinguals. Recent reviews and expert commentaries have called for more detailed characterisation of bilingual participants, recommending the use of standardised, objective proficiency measures and reporting of individual bilingual language histories [85,103,104]. Adopting a continuum-based perspective would allow researchers to disentangle the specific aspects of bilingualism that contribute to executive function development more precisely, and may help elucidate some of the current variability in empirical results [105,106,107].

It is important to note that none of the included studies employed validated bilingual profiling tools, limiting the depth of understanding of the relationship between bilingualism and EF. Future studies could employ validated bilingualism profiling tools such as the Language Experience and Proficiency Questionnaire [108] or the bilingual language profile [109]. Standard reporting checklists may also be considered such as those that include (i) the amount of bilingual exposure, (ii) the age of acquisition for each language, (iii) the current frequency and context of language use, (iv) language switching patterns in daily life, (v) objective language proficiency measures for both languages, and (vi) home language environment characteristics. These measures could be used to provide a comprehensive characterisation of participants’ bilingual experiences and facilitate a better understanding of the relationships between bilingualism and cognitive development.

### 4.4. Clinical and Educational Implications

The findings from this review are limited to children with ASD but may offer preliminary evidence for consideration in clinical practice and educational policy in relation to the longstanding concerns about bilingual exposure in children with NDDs. The evidence suggesting that bilingualism is associated with better EF performance in children with ASD contributes to the discussions that address traditional concerns about the potential negative effects of bilingualism in NDD populations [110]. The findings of this scoping review are specific to children with ASD, suggesting that current evidence runs counter to the concern of parents and professionals that exposure to more than one language increases the burden on children with NDDs.

For families of children with ASD who are considering bilingual education, these findings provide preliminary evidence that bilingual exposure may not be burdensome but instead could potentially have associated benefits for specific domains of EF development. However, given the heterogeneity of results and the evidence drawn from cross-sectional studies, individualised approaches remain essential with careful consideration of each child’s unique strengths and challenges.

### 4.5. Limitations and Future Research Directions

Several limitations of the current evidence base require consideration. The exclusive focus on ASD limits our understanding of the relationship between bilingualism and EF across other NDD groups. EF impairments are transdiagnostic and affect children with DCD, intellectual disabilities, ADHD, and other NDDs [31]. Moreover, high rates of comorbidity exist between these conditions, with many children presenting with multiple NDDs simultaneously [29]. This comorbidity pattern suggests that research focusing on different NDDs may reveal key knowledge missing from reviews examining specific NDDs in isolation. Our understanding of the inter-relationships between these conditions and how bilingualism might address shared underlying deficits is currently insufficient. Future research is therefore warranted to explore the potential contribution of bilingualism in addressing the needs of children across the NDD spectrum. This is particularly important because each NDD is unique in its clinical presentation while sharing common impairments in cognition, communication, behaviour, and/or motor skills [111]. This presents an important research direction that may be pursued through observational and intervention studies.

The restriction to English-language publications, noted in Section 2, represents an additional limitation that may have excluded relevant studies in other languages. The variability in assessment methods and the discrepancies between different measurement approaches highlight the need for more standardised assessment protocols. Future research should use multi-method approaches that combine performance-based tasks with parent-reported measures to provide a more comprehensive understanding of EF outcomes as they relate to bilingualism. The inconsistent control for confounders, such as gender, SES, and intelligence, across studies represents a significant limitation that may contribute to variability in findings and limit comparability. Future research should prioritise consistent and comprehensive control of these confounders to enhance the reliability of the results.

Another important limitation concerns the factor of linguistic distance, which refers to the degree of difference between two languages or dialects [112]. We found no study that provided relevant data on linguistic distance; hence, this review cannot provide an evidence base for investigating the role of linguistic distance in the apparent bilingual cognitive advantages among children with ASD. This critical gap warrants consideration in future research. A recent study by Gallo et al. [113] suggested that linguistic distance may modulate bilingual effects on EF. They demonstrated that more distant language pairs (such as those differing substantially in grammar, phonology, and writing systems) exerted maximum cognitive effects at the initial stages of bilingual experience. On the other hand, closer language pairs had greater impacts at more advanced stages of bilingual development. This finding has important implications for understanding bilingual-related differences in children with ASD, as the cognitive benefits of bilingualism may vary depending on the specific language combinations to which children are exposed. Future studies should examine how linguistic distance influences EF outcomes in children with ASD, considering that different language pairs may place varying demands on domain-general cognitive processes and thus potentially yield different patterns of cognitive benefits [113]. Future research could include linguistic distance as a reporting variable to test its moderating role in bilingual cognitive effects. This approach would enable researchers to measure the impact of linguistic distance on EF outcomes and better understand its influence across ASD and other NDD populations and language combinations.

Additionally, the mechanisms underlying bilingual-related differences in EF across children with ASD and those with typical development remain poorly understood. Cognitive processes in managing two languages may be viewed as theoretical explanations of the benefits of EF outcomes. However, future research should generate empirical evidence and investigate potential mediating factors, such as language proficiency, age of acquisition, and intensity of bilingual exposure, to better understand how and why bilingual advantages emerge. This mechanistic understanding forms a crucial piece of evidence for clinicians considering bilingual approaches as part of intervention programmes for children with ASD.

## 5. Conclusions

This scoping review provides the first comprehensive synthesis of research examining the relationship between bilingualism and EF outcomes in children with NDDs, revealing a generally positive but complex picture exclusively focused on ASD. The evidence suggests that bilingual exposure is associated with specific cognitive benefits in children with ASD, particularly in working memory updating processes, certain aspects of inhibitory control, and cognitive flexibility. The most significant findings emerged in performance-based tasks that include the two-back task for working memory and the DCCS for set-shifting abilities, where bilingual children with ASD, in some cases, performed comparably better than TD children. It is important to emphasise that this evidence is specific to ASD and cannot currently be generalised to other NDDs due to the absence of research in these populations. The methodological diversity across studies revealed important discrepancies between performance-based tasks and parent-reported measures, suggesting that bilingual advantages may be context-dependent. These findings contribute to evidence questioning longstanding concerns about the potential negative effects of bilingual exposure in NDD populations and provide encouraging evidence for families considering bilingual education for their children with ASD.

The exclusive focus of the available evidence on ASD represents both a limitation that constrains generalisability across the broader spectrum of NDDs and a research gap that future research should address. Studies beyond ASD need to examine the role of bilingualism in EF development across the range of NDDs, while employing more comprehensive assessment approaches that combine performance-based tasks with parent-reported measures. Finally, functional EF assessments in naturalistic settings (e.g., home and school environment) may be considered to address the discrepancy between laboratory-based and parent-reported findings highlighted in this review.

## Figures and Tables

**Table 1:** Executive function difficulties among NDD conditions across domains.

NDD	Inhibitory Control	Working Memory	Cognitive Flexibility
ASD [13,14,15]	√	√	√
DCD [12]	√	√	
CP [16,17]	√	√	
ADHD [19,20]	√	√	√
ID [18]	√	√	√

**Table 2:** Summary of the participants, settings, and EF assessments for all studies.

Participants	Setting	EF Domains	EF Assessments
Age: 5–12 years old (n = 15) Groups: Monolingual ASD (n = 463) Bilingual ASD (n = 404) Monolingual TD (n = 346) Bilingual TD (n = 298)	Greece (n = 6) Canada (n = 6) United States (n = 3) United Arab Emirates (n = 2) Japan (n = 1)	Inhibition	Global–local tasks (n = 4)
			Simon task (n = 3)
			Go/No-Go task (n = 1)
			Stroop task (n = 1)
			Flanker task (n = 1)
		Set shifting	Dimensional Change Card Sort (DCCS) (n = 2)
			Wisconsin Card-Sorting Task (WCST) (n = 1)
		Working memory	Two-back task (n = 6)
			Listening span task (n = 1)
			Number repetition (n = 1)
			Self-Ordered Pointing Task (SOPT) (n = 1)
			Digit span (n = 1)
		Multidomain	Behaviour Rating Inventory of Executive Function (BRIEF) (n = 4)
			Comprehensive Executive Function Inventory (CEFI) (n = 2)

**Table 3:** Summary of the outcomes by executive function (EF) domains.

EF Domain	Study	Outcomes
Working memory (performance-based tasks)	- Andreou et al., 2020 [54] - Baldimtsi et al., 2020 [55] - Peristeri et al., 2020 [56] - Peristeri et al., 2021a [57]	Bilingual children with ASD showed higher accuracy and faster response times compared to monolingual peers.
	- Barrero 2017 [58] - Li et al., 2017 [59] - Sharaan, FletcherWatson & MacPherson 2021 [60] - Siroski 2017 [61]	No significant differences were found between monolingual and bilingual ASD groups.
Working memory (parent-reported)	- Iarocci, Hutchison & O’Toole 2017 [62] - Ratto, Reimann & Nadwodny 2022 [63] - Sharaan, MacPherson & FletcherWatson 2022 [64]	Bilingual children with ASD had fewer reported working memory problems and showed lower impairment in self-monitoring.
	- Macaro 2015 [65]	Results showed no significant differences in working memory.
Inhibition/interference control (performance-based tasks)	- Baldimtsi et al., 2020 [55] - Li et al., 2017 [59] - Peristeri et al., 2020 [56] - Peristeri et al., 2021a [57]	Bilingual children showed faster response times for incongruent trials, and they also showed better cognitive control.
	- Li et al. 2017 [59] - Sharaan, FletcherWatson & MacPherson 2021 [60] - Siroski 2017 [61]	Other performance-based measures, like the Simon task and Flanker task, sometimes showed no significant differences between monolingual and bilingual ASD groups.
Inhibition/interference control (parent-reported)	- Romero et al., 2023 [66] - Sharaan, MacPherson & FletcherWatson 2022 [64]	Bilingual children with ASD had lower reported inhibition difficulties compared to monolingual peers.
Shifting/cognitive flexibility (performance-based tasks)	- Barrero 2017 [58] - Peristeri et al., 2021a [57] - Peristeri et al., 2021b [67] - Romero et al., 2023 [66]	Bilingual children with ASD showed significant advantages in set-shifting tasks compared to monolingual peers, with bilingual ASD children performing similarly to their TD peers.
Shifting/cognitive flexibility (parent-reported)	- Ratto, Reimann & Nadwodny 2022 [63] - Romero et al., 2023 [66]	Results showed lower parent-reported problems with shifting for bilingual children with ASD.
	- Barrero 2017 [58] - Labonté 2022 [68] - Macaro 2015 [65] - Sharaan, FletcherWatson & MacPherson 2021 [60] - Sharaan, MacPherson & FletcherWatson 2022 [64] - Siroski 2017 [61]	Parental reports did not show a significant advantage for bilingual children with ASD in flexible switching.

## Data Availability

Not applicable.

## Supplementary Materials

The following supporting information can be downloaded at: https://www.mdpi.com/article/10.3390/children12091247/s1. Table S1. Summary table of studies included in the review.
