# Supplementary material for: Effects of Bilingualism on Executive Function of Children with Neurodevelopmental Disorders: A Scoping Review
Source: Children (Basel). 2025 Sep 17;12(9):1247. doi: 10.3390/children12091247 (PMC12468967; doi:10.3390/children12091247)
Supplement: Supplementary file 1 [file children-12-01247-s001.zip › children-3810875-supplementary.pdf]

Table S1. Summary table of studies included in the review.

Study	Setting, study design	Participants	Outcome measures (EF)	Outcome measures (Language)	Results
Andreou et al. (2020) [54]	**Setting:** Greece<br><br>**Study design:** Cross sectional	**Total number of participants: 56**<br><br>**Inclusion/exclusion criteria:**<br>- Normal IQ<br>- High functioning autism<br>- Responded correctly to reality control questions<br><br>**Diagnosis:** ASD<br><br>**Monolingual group:**<br>*ASD*<br>- n=27<br>- Gender (male/female): 18/9<br>- Age: 10;04 ± 1.8<br><br>**Bilingual Group:**<br>*ASD*<br>- n=29<br>- Gender (male/female): 20/9<br>- Age: 10;04 ± 1.7	- Two-back working memory and updating task<br>- Theory of Mind (ToM):<br>▫ Sally-Anne task<br>▫ Unexpected contents task<br>▫ First-order false belief (FB) task	- Expressive vocabulary in Greek<br>- Sentence repetition task	Bilingual children with ASD outperformed monolingual children in low-verbal ToM and EF tasks, with bilinguals' ToM performance linked to EF and adverbial clause repetition, while monolinguals' verbal ToM performance was associated with complement syntax, indicating distinct mentalising pathways.

Baldimtsi et al. (2020) [55]	**Setting**: Greece<br><br>**Study design**: Cross sectional	**Total number of participants:** 136<br><br>**Inclusion/exclusion criteria:**<br>*TD*<br>- Normal hearing<br>- No emotional, mental, neurodevelopmental or language impairment<br>*ASD*<br>- With diagnosis<br>- Non-verbal IQ of at least 70<br>- No history of language delay<br><br>**Diagnosis:** ASD<br><br>**Monolingual group:**<br>*TD*<br>- n=35<br>- Gender (male/female): NA<br>- Age: 10;07 ± 1.6<br>*ASD*<br>- n=34<br>- Gender (male/female): NA	- Online visual attention-switching global-local task<br>- Two-back task<br>- ToM:<br>▫ False-belief task		Bilingual children with and without ASD outperformed their monolingual peers in low-verbal ToM and EF tasks, including visual attention switching and updating, suggesting that bilingualism enhances mentalising and cognitive control, potentially due to improved inhibitory control, with bilingual ASD children showing performance closer to TD peers.

		- Age: 11;07 ± 2.0<br><br>**Bilingual Group:**<br>*TD*<br>- n=34<br>- Gender (male/female): NA<br>- Age: 11;01 ± 1.7<br>*ASD*<br>- n=34<br>- Gender (male/female): NA<br>- Age: 11;06 ± 1.4			
Peristeri et al. (2020) [56]	**Setting**: Greece<br><br>**Study design**: Cross sectional	**Total number of participants:** 80<br><br>**Inclusion/exclusion criteria:**<br>- IQ scores above 70<br><br>**Diagnosis:** ASD<br><br>**Monolingual group:**<br>*TD*<br>- n=20<br>- Gender (male/female): 20/0<br>- Age: 9;07 ± 1.1<br>*ASD*	- Two-back test<br>- Visual attention global-local task	- Expressive vocabulary task<br>- Sentence repetition task	Bilingual children with ASD outperformed their monolingual peers in both macrostructural (story structure complexity and fewer ambiguous referential expressions) and microstructural (higher adverbial clause use) aspects of narrative production, with enhanced executive function (EF) skills, particularly in global attention and updating, contributing to their superior narrative performance, despite no significant differences in

		- n=20<br>- Gender (male/female): 20/0<br>- Age: 9;08 ± 1.5<br><br>**Bilingual Group:**<br>*TD*<br>- n=20<br>- Gender (male/female): 20/0<br>- Age: 9;08 ± 1.1<br>*ASD*<br>- n=20<br>- Gender (male/female): 20/0<br>- Age: 9;07 ± 1.3			general cognitive or language abilities.
Peristeri et al. (2021a) [57]	**Setting**: Greece<br><br>**Study design**: Cross sectional	**Total number of participants:** 103<br><br>**Inclusion/exclusion criteria:**<br>-<br><br>**Diagnosis:** ASD<br><br>**Monolingual group:**<br>*ASD*<br>- n=60<br>- Gender (male/female):	- WISC-III<br>- Two-back task<br>- Visual attention global-local task<br>- ToM: first order FB task	- Expressive vocabulary task<br>- Sentence repetition task	Bilingual children with ASD outperformed their monolingual peers in first-order FB reasoning and EF tasks, particularly in working memory, updating, and cognitive flexibility, despite lower expressive vocabulary, suggesting bilingualism enhances FB understanding and EF without negatively impacting linguistic development.

		49/11<br>- Age: 11.43 ± 2.14<br><br>**Bilingual Group:**<br>*ASD*<br>- n=43<br>- Gender (male/female): 35/8<br>- Age: 11.56 ± 1.75			
Ratto, Reimann & Nadwodny (2022) [63]	**Setting**: USA<br><br>**Study design**: Cross sectional	**Total number of participants:** 159<br><br>**Inclusion/exclusion criteria:**<br>- Children with unclear language exposure histories<br><br>**Diagnosis:** ASD<br><br>**Monolingual group:**<br>*ASD*<br>- n=53<br>- Gender (male/female): 45/8<br>- Age: 9.6 ± 2.26<br><br>**Bilingual Group:**	- Wechsler Abbreviated Scale of Intelligence Second Edition (WASI-II)<br>- Wechsler Adult Intelligence Scale Third Edition (WAIS-III)<br>- WISC-IV<br>- WISC-V<br>- BRIEF		Dual-language learning (DLL) in autistic youth was associated with lower verbal IQ, but it positively impacted parent-reported EF skills, particularly in flexibility, working memory, and metacognitive abilities like organisation and self-monitoring, while also showing fewer repetitive behaviors compared to monolingual peers, suggesting potential benefits of DLL.

		ASD<br>- n=106<br>- Gender (male/female): 90/16<br>- Age: 9.7 ± 2.25			
Iarocci, Hutchison & O'Toole (2017) [62]	**Setting**: Canada<br><br>**Study design**: Cross sectional	**Total number of participants:** 109<br><br>**Inclusion/exclusion criteria:**<br>- No intellectual disability<br><br>**Diagnosis:** ASD<br><br>**Monolingual group:**<br>*TD*<br>- n=24<br>- Gender (male/female): 10/14<br>- Age: 9;27 ± 1.56<br>*ASD*<br>- n=52<br>- Gender (male/female): 43/9<br>- Age: 10;08 ± 1.94<br><br>**Bilingual Group:**	- Behavior Assessment System for Children Second Edition (BASC-2)	- BASC-2	Second language exposure in children with ASD does not delay cognitive or functional communication skills and is associated with a lower percentage of clinically significant EF and FC impairments compared to non-exposed peers.

		TD<br>- n=59<br>- Gender (male/female): 35/24<br>- Age: 9;71 ± 1.76<br>*ASD*<br>- n=39<br>- Gender (male/female): 32/7<br>Age: 10;21 ± 2.47			
Barrero (2017) [58]	**Manuscript 3**<br><br>**Setting**: Canada<br><br>**Study design**: Cross sectional	**Total number of participants:** 40<br><br>**Inclusion/exclusion criteria:**<br>- Normal IQ<br>- No acute medical conditions<br>- No history of language, learning or developmental difficulties, physical, visual or hearing limitations, no family members with ASD<br><br>**Diagnosis:** ASD<br><br>**Monolingual group:**	- Behavior Rating Inventory of Executive Function (BRIEF) parent form<br>- Dimensional change card sort task (DCCS)<br>- Forward and backward digit span	- Peabody Picture Vocabulary Test Fourth Edition (PPVT-4)<br>- Recalling sentences subtest from Clinical Evaluation of Language Fundamentals Fourth Edition (CELF-IV)	Bilingual children, both with and without ASD, surpassed monolingual peers in low-verbal ToM and EF tasks like visual attention switching and updating, indicating bilingualism boosts mentalizing and cognitive control, likely via enhanced inhibitory control, with bilingual ASD children performing more like TD peers.

		TD<br>- n=10<br>- Gender (male/female): 7/3<br>- Age: 7;09 ± 0.63<br>*ASD*<br>- n=10<br>- Gender (male/female): 10/0<br>- Age: 8;04 ± 0.995<br><br>**Bilingual Group:**<br>*TD*<br>- n=10<br>- Gender (male/female): 7/3<br>- Age: 7;11 ± 0.744<br>*ASD*<br>- n=10<br>- Gender (male/female): 8/2<br>- Age: 8;01 ± 0.603			
Li et al. (2017) [59]	**Setting**: Japan<br><br>**Study design**: Cross sectional	**Total number of participants:** 67<br><br>**Inclusion/exclusion criteria:**<br>- No intellectual disability<br><br>**Diagnosis:** ASD	- Raven Coloured Progressive Matrices<br>- Stroop task<br>- Simon Task<br>- Go/No-Go Task<br>- Wisconsin Card Sorting Test (WCST)	- PPVT-4	No significant differences in EF, communication, or social behavioral skills between bilingual and Japanese monolingual children with ASD, except for faster performance by bilingual ASD children on the

		**Monolingual group:**<br>*TD*<br>- n=15<br>- Gender (male/female): 11/4<br>- Age: 8;11 ± 1.761<br>*ASD*<br>- n=19<br>- Gender (male/female): 15/4<br>- Age: 8;04 ± 1.467<br><br>**Bilingual Group:**<br>*TD*<br>- n=20<br>- Gender (male/female): 16/4<br>- Age: 8;04 ± 1.12<br>*ASD*<br>- n=13<br>- Gender (male/female): 11/2<br>- Age: 9;02 ± 1.822		Stroop task, suggesting no bilingual advantage or impairment, though results are limited by small sample size, wide age range, task difficulty, and cultural and testing environment variations.
Sharaan, Fletcher-Watson & MacPherson (2021) [60]	**Setting**: UK<br><br>**Study design**: Cross sectional	**Total number of participants:** 93<br><br>**Inclusion/exclusion criteria:**<br>- Children with formal ASD	- DCCS task<br>- Simon task<br>- Psychomotor Vigilance Task (PVT)<br>- Self-Ordered Pointing	The study found no widespread EF advantages from bilingualism in autistic or TD children, except for a preliminary advantage in sustained attention for bilingual

		diagnosis<br><br>**Diagnosis:** ASD<br><br>**Monolingual group:**<br>*TD*<br>- n=32<br>- Gender (male/female): 13/19<br>- Age: 111.03 ± 27.74<br>*ASD*<br>- n=10<br>- Gender (male/female): 8/2<br>- Age: 99.11 ± 22.78<br><br>**Bilingual Group:**<br>*TD*<br>- n=34<br>- Gender (male/female): 8/26<br>- Age: 110.50 ± 19.63<br>*ASD*<br>- n=17<br>- Gender (male/female): 10/7<br>- Age: 115.56 ± 24.24	Task (SOPT)		autistic children, who made fewer false starts, though autistic children generally performed worse in EF tasks, and bilingualism did not negatively impact their EF abilities.
Siroski (2017)	**Setting**: Canada	**Total number of participants:**	- The Kaufman Brief	- PPVT	Among ten children with ASD,

[61]	**Study design**: Cross sectional	10<br><br>**Inclusion/exclusion criteria:**<br>- Children with formal ASD diagnosis<br>- Between 6-10 years of age<br>- Exposed to only English or English plus another language on a regular basis<br>- No intellectual disability, genetic or chromosomal anomaly, or significant and/or uncorrected vision or hearing impairment<br><br>**Diagnosis:** ASD<br><br>**Monolingual group:**<br>*ASD*<br>- n=4<br>- Gender (male/female): 2/2<br>- Age: 107<br><br>**Bilingual Group:**<br>*ASD*	Intelligence Test (KBIT)<br>- Simon task<br>- Flanker task<br>- Forward and backward digit span working memory task		regular physical fitness was the only activity associated with better performance in EF tasks involving inhibition and visuospatial working memory, while dual language exposure, video game playing, and music lessons showed no significant EF benefits, though results are limited by the small sample size.

		- n=6<br>- Gender (male/female): 6/0<br>- Age: 103.67			
Macaro (2015) [65]	**Setting**: Canada<br><br>**Study design**: Cross sectional	**Total number of participants:** 67<br><br>**Inclusion/exclusion criteria:**<br>- Participants between 8;05-9;00 who had completed all measures<br><br>**Diagnosis:** ASD<br><br>**Monolingual group:**<br>*ASD*<br>- n=21<br>- Gender (male/female): 17/4<br>- Age: 8.663 ± 0.159<br><br>**Bilingual Group:**<br>*ASD*<br>- n=21<br>- Gender (male/female): 18/3<br>- Age: 8.758 ± 0.232	- BRIEF<br>- Wechsler Individual Achievement Test Second Edition (WIAT-II)<br>- Wechsler Intelligence Scale for Children Fourth Edition (WISC-IV)	- CELF-IV	No significant differences in EF or academic achievement (math and reading) between monolingual and bilingual children with ASD, with bilingual children showing a non-significant tendency to perform better on most EF scales and academic tests, but fewer correlations between EF and academic achievement compared to the monolingual group, suggesting bilingualism has no negative impact and may interact differently with EF and academic skills in ASD.
Romero et al.	**Setting**: USA	**Total number of participants:**	- BRIEF		Bilingual children with ASD

(2023) [66]	**Study design**: Cross sectional	112<br><br>**Inclusion/exclusion criteria:**<br>- Children with no core ASD symptoms<br><br>**Diagnosis:** ASD<br><br>**Monolingual group:**<br>*TD*<br>- n=25<br>- Gender (male/female): 15/11 *wrong number on original study<br>- Age: 9.90 ± 1.61<br>*ASD*<br>- n=31<br>- Gender (male/female): 25/6<br>- Age: 10.14 ± 1.63<br><br>**Bilingual Group:**<br>*TD*<br>- n=35<br>- Gender (male/female): 26/9<br>- Age: 9.96 ± 1.61		exhibited stronger EF skills, particularly in inhibition and shifting, and enhanced perspective-taking abilities compared to their monolingual peers, with bilingualism indirectly reducing core ASD symptoms like social communication deficits and repetitive behaviors through improved EF.

		ASD<br>- n=21<br>- Gender (male/female): 20/1<br>- Age: 9.75 ± 1.60		
Sharaan, MacPherson & Fletcher-Watson (2022) [64]	**Setting**: UK<br><br>**Study design**: Cross sectional	**Total number of participants:** 80<br><br>**Inclusion/exclusion criteria:**<br>- Children with formal ASD diagnosis<br><br>**Diagnosis:** ASD<br><br>**Monolingual group:**<br>*TD*<br>- n=29<br>- Gender (male/female): 6/23<br>- Age: 114.48 ± 25.98<br>*ASD*<br>- n=21<br>- Gender (male/female): 17/4<br>- Age: 104.76 ± 23.90<br><br>**Bilingual Group:**<br>*TD*	- CEFI	Bilingual autistic children showed advantages in parent-reported EF skills, including interference control, flexible switching, sustained attention, and unexpectedly, working memory, compared to their monolingual autistic peers, despite no such advantages in teacher reports or for TD children, indicating bilingualism does not harm EF in autistic children and may mitigate some EF difficulties in a dual-language context.

		- n=24<br>- Gender (male/female): 11/13<br>- Age: 110.08 ± 19.54<br>*ASD*<br>- n=6<br>- Gender (male/female): 4/2<br>- Age: 116.17 ± 16.31			
Peristeri et al. (2021b) [67]	**Setting**: Greece<br><br>**Study design**: Cross sectional	**Total number of participants:** 200<br><br>**Inclusion/exclusion criteria:** NA<br><br>**Diagnosis:** ASD<br><br>**Monolingual group:**<br>*TD*<br>- n=50<br>- Gender (male/female): 38/12<br>- Age: 9.68 ± 0.94<br>*ASD*<br>- n=50<br>- Gender (male/female):	- Global-local cognitive flexibility<br>- Dual-task paradigms<br>▫ Listening span task<br>▫ Syntactic interference word recall task<br>▫ Proactive interference task	- Expressive vocabulary<br>- Sentence repetition	Bilingual autistic children outperformed their monolingual peers in word recall across three dual-task paradigms (listening span, syntactic interference, and proactive interference), demonstrating that bilingualism mitigates cognitive flexibility deficits in autism, primarily driven by enhanced EF skills rather than language abilities.

		40/10<br>- Age: 9.94 ± 1.46<br><br>**Bilingual Group:**<br>*TD*<br>- n=50<br>- Gender (male/female): 40/10<br>- Age: 9.84 ± 1.11<br>*ASD*<br>- n=50<br>- Gender (male/female): 38/12<br>- Age: 9.66 ± 1.61			
Labonté (2022) [68]	**Second study**<br><br>**Setting**: Canada<br><br>**Study design**: Cross sectional	**Total number of participants:** 109<br><br>**Inclusion/exclusion criteria:** NA<br><br>**Diagnosis:** ASD<br><br>**Monolingual group:**<br>*TD*<br>- n=17	- BRIEF<br>- Comprehensive Executive Function Inventory (CEFI)	- Child Language Exposure Questionnaire<br>- Alberta Language and Development Questionnaire (ALDeQ)	Bilingualism does not disadvantage the development of autistic children, with no adverse effects on parent-reported EF and adaptive functioning across diverse language exposure conditions, supporting the notion that bilingualism is beneficial and countering recommendations against it for autistic children.

- Gender (male/female): 9/8
- Age: 8.20 ± 2.986

ASD

- n=17
- Gender (male/female): 16/1
- Age: 10.98 ± 2.215

Bilingual Group:

TD (simultaneous)

- n=22
- Gender (male/female): 9/13
- Age: 8.85 ± 3.112

TD (sequential)

- n=16
- Gender (male/female): 10/6
- Age: 9.52 ± 3.198

ASD (simultaneous)

- n=21
- Gender (male/female): 15/6
- Age: 9.91 ± 3.00

ASD (sequential)

- n=16
- Gender (male/female): 11/5
- Age: 9.66 ± 2.936
